# Dynamics of Cooperation in a Task Completion Social Dilemma

**DOI:** 10.1371/journal.pone.0170604

**Published:** 2017-01-26

**Authors:** Luis Felipe Giraldo, Kevin M. Passino

**Affiliations:** 1 Department of Electrical and Electronics Engineering, Universidad de Los Andes, Bogotá, Colombia; 2 Department of Electrical and Computer Engineering, The Ohio State University, Columbus, Ohio, United States of America; Tianjin University of Technology, CHINA

## Abstract

We study the situation where the members of a community have the choice to participate in the completion of a common task. The process of completing the task involves only costs and no benefits to the individuals that participate in this process. However, completing the task results in changes that significantly benefit the community and that exceed the participation efforts. A task completion social dilemma arises when the short-term participation costs dissipate any interest in the community members to contribute to the task completion process and therefore to obtain the benefits that result from completing the task. In this work, we model the task completion problem using a dynamical system that characterizes the participation dynamics in the community and the task completion process. We show how this model naturally allows for the incorporation of several mechanisms that facilitate the emergence of cooperation and that have been studied in previous research on social dilemmas, including communication across a network, and indirect reciprocity through relative reputation. We provide mathematical analyses and computer simulations to study the qualitative properties of the participation dynamics in the community for different scenarios.

## Introduction

Human cooperation is the process of people acting collectively toward a common end. People who decide to cooperate usually have to pay costs associated with their individual contribution during this process, but this can result in them gaining greater benefits, ones that result from collective coordination and action [1, 2]. A social dilemma is the situation that arises when the individual interests are not aligned with the collective ones. The costs and problems involved in cooperation can make individuals in this dilemma behave in a way that they opt not to participate in the collective action. In this case, “individual rationality leads to collective irrationality” ([3],p 183). It is very important then to understand the conditions that cause social dilemmas, and how to motivate people to cooperate and enjoy the benefits that result from their collective action.

Many important situations have the characteristics of a social dilemma. For example, the situation where individuals can decide to commute by either automobile or public transportation. Commuting by automobile might provide individual benefits that include shorter travel times and flexibility. However, as more individuals choose this option, consequences such as air pollution and traffic congestion increase as well, negatively affecting the whole community [4]. The social dilemma arises when the individual interests are in favor of maximizing their own immediate benefits, such as travel convenience, leading to decisions that are detrimental to the community. Similar situations occur in other domains and scales, such as household energy consumption and conservation [5], provision in the welfare state [6], and the relation between our attitude toward nature and dangerous climate change [7, 8]. The common pattern that characterizes these cases is that the shortsighted actions of the individuals prevent the entire community from obtaining long-term benefits.

In this work, we study those situations where the individuals in a community have the choice to contribute toward the completion of a common task. The process of task completion might involve only costs and no immediate benefits to the community members while they are participating. However, the completion of the task results in changes that substantially benefit the community, including all its members, and that exceed the participation efforts. An interesting situation that conceptually exemplifies this is “barn raising” ([9],Ch 6). This practice entails building a barn that will be either owned by an individual or shared by the community. The individuals who decide to collaborate do not get any payment, and the process of completing the task can be time consuming and requires a significant effort. Each individual’s degree of involvement cannot be arbitrarily large, but the individuals that are involved in a task completion process have to distribute the available work load between them. The result of completing the task, that in this case is building the barn, includes the functional benefits provided by the barn in the community and the strengthening of the social bonds between the community members. We do not focus on studying the development of trust as the result of the iterated completion of tasks, as has been studied before [10]. We focus our analysis on the process of promoting participation and engaging people in the community to complete the task.

Even though the results of completing the task can be highly desirable, self-interested individuals easily turn this situation into a social dilemma: the short-term costs associated with participation can dissipate any motivation to contribute to the completion of the task and therefore to obtain the benefits that it provides in the long run. We call this situation a *task completion social dilemma*. Some of the basic features that characterize the task completion problem are:

- The community does not get benefits until the task is completed.
- Each individual knows the current state of the task.
- The task is completed by the continued contributions from the individuals in the community.
- Each individual pays participation costs during the process of task completion.

This problem is similar to the public goods problem [11–13] in that individuals are able contribute an amount of resources to generate a collective good for the benefit of the whole community by paying costs associated with their individual contribution. However, the facts that the benefits of the collective good will be available only when the task has been completed, and that the community knows the current progress of the task that is being completed, are characteristics that, taken together, differentiate a task completion problem from the other types of problems studied in the context of social dilemmas.

Due to their importance, a large body of research has been conducted to understand different aspects of cooperation in social dilemmas. Work in several disciplines ranging from evolutionary biology [14–17], [18] and ecology [19–21] to social phycology [11, 12, 22, 23] and political science [24, 25], has provided models and theoretical insights that try to explain the conflict between the individual and the group interests, and the mechanisms that allow for the emergence of cooperation. Inspired by some of these developments, we present in this paper a mathematical model that captures the relationship between the task to be completed and the contribution provided by the individuals in the community. We propose basic mechanisms that promote participation and cooperation during a task completion process. This relationship, and the ways to promote active participation by the community members, represent a complex problem [9, 26, 27], where the different factors to be considered can grow considerably in number. However, our work focuses on those elements that we have identified to be the keys to accurately describe and study a task completion social dilemma, and the conditions that are suitable for the emergence of cooperation.

Different mathematical models have been proposed to study cooperation, employing concepts commonly used in game theory [14, 28–30], theory of dynamical systems [31, 32], and optimization [12, 33]. The metaphorical use of these models has been shown to be helpful in the analysis of the conditions that promote collective action and cooperation ([14],Ch 1),([24],Ch 1),([26],Ch 2). In the same spirit, due to the nature of the problem, we characterize and analyze the process of task completion as a discrete dynamical system. In this model, the task is completed by the iterated contribution of the community members to the task. We refer to “participation load” as the degree of involvement of the individual in the task. Then, given his/her participation load, the contribution of an individual to the completion of the task is characterized by a *production function*. This function has been previously used in the analysis of collective action and common goods to describe the relationship between the resources provided by the individual and the amount of collective good that is generated by those resources ([12],Ch 4), [28]. In our problem, the production function is used to model the relationship between the participation load and the contribution of this load to the completion of the task. The dynamics of participation are modeled to include mechanisms that promote participation in a way that the individuals share their participation load and costs between them. We incorporate into our model the concept of *motivation* as the willingness of the individual to assume such costs by participating in the task completion process. In the context of social dilemmas, experiments have been conducted in human groups to study the different factors and situations that cause motivation gain or loss during the process of participation in a task when the individuals are required to work either individually or collectively [22, 34–38]. Second, we model the community so that the individuals are able to communicate with each other via an interaction network. It has been shown that *communication* is crucial for the emergence of cooperation [3, 39, 40]. In our dynamical model, communication allows an individual to build its *relative reputation*, defined as the reputation of an individual from the viewpoint of another one, as a mean to promote participation in the community through reciprocity [19, 41]. Also, the ability to *locally* interact enables the individuals to dynamically distribute the participation load between them based on the individual costs of participation and their motivation.

The mathematical model that we propose can be seen as an extension of the work in [12] in the context of collective goods. The authors in [12] model the gain of a community member based on the benefits obtained by the current level of the collective good and the costs associated with the individual contributions, and study the effect of different forms of production functions on the generation of the collective good. Although their work is seminal in the study of cooperation and social dilemmas, they recognize that their models are essentially static and that there is a need for the development of dynamical models that build on their work ([12],p. 190). In our work, we formulate a dynamical system that characterizes the evolution of the task completion process as the result of the repeated contribution of the individuals in the community based on their motivation and participation costs, allowing us to study mechanisms that promote participation and cooperation in the community. We use concepts of stability analysis of discrete nonlinear systems and Monte Carlo simulations to analyze the qualitative and quantitative behavior of the modeled community. The paper closes with a discussion on the metaphorical use of the model and future developments of the model.

## Model Formulation

### Task Completion Dynamics

The amount of participation load taken by an individual to help solve the task is denoted by $p_{i}\in R_{\geq 0}=\left[0,\infty \right)$. We assume that the task to be completed allows the community to take a participation load up to *P* > 0. It means that the individuals cannot take an arbitrary participation load, but it must be distributed so that

$$\sum_{i=0}^{n}p_{i}\leq P$$ (1)

where *n* ≥ 1 is the number of individuals.

The contribution of the all individuals toward the completion of the task, given the participation load vector *p* = [*p*_1_, …, *p*_*n*_]^⊤^, is characterized by the *production function* $h\left(p\right):R_{\geq 0}^{n}\to R_{\geq 0}$. Let *z* ≥ 0 be the variable that quantifies the task completion level. A value of *z* = 0 indicates that the task has not been started, and $z=\bar{z}>0$ indicates that the task has been completed. The dynamics of task completion describe how *z* changes depending on the individuals’ participation patterns, and their contribution described by the production function. We characterize the dynamics of task completion via

$$z\left(t+1\right)=z\left(t\right)+\phi h\left(p(t)\right)\left(\bar{z}-z\left(t\right)\right)$$ (2)

where *z*(*t*) and *p*(*t*) are the task completion and participation load variables at time step t∈N, and *ϕ* ∈ (0, 1] is a parameter that scales the action of the production function on the completion of the task. Note that the production function *h*(*p*) drives the rate of change of the task variable. According to this equation, the task is at an *equilibrium point* when either the individuals’ participation pattern is such that there is no contribution to complete the task, or the task has been already completed. In other words, we have that *z*(*t* + 1) = *z*(*t*) when either *h*(*p*(*t*)) = 0 or $z\left(t\right)=\bar{z}$.

Different forms of the production function can be used to capture different situations in the relationship between the participation load and its contribution to the task completion. In general, we assume that the production function: (i) is the result of the additive contribution of each individual, (ii) is monotonic increasing with respect to each individual’s participation load, and (iii) satisfies *h*(0) = 0. This means that the contribution to the completion of the task increases as the individuals increase their participation, and there is no contribution if no individual in the community takes any participation load. These assumptions on the production function imply that *h*(*p*(*t*)) ≥ 0 for all *t* ≥ 0.

**Fig 1:**
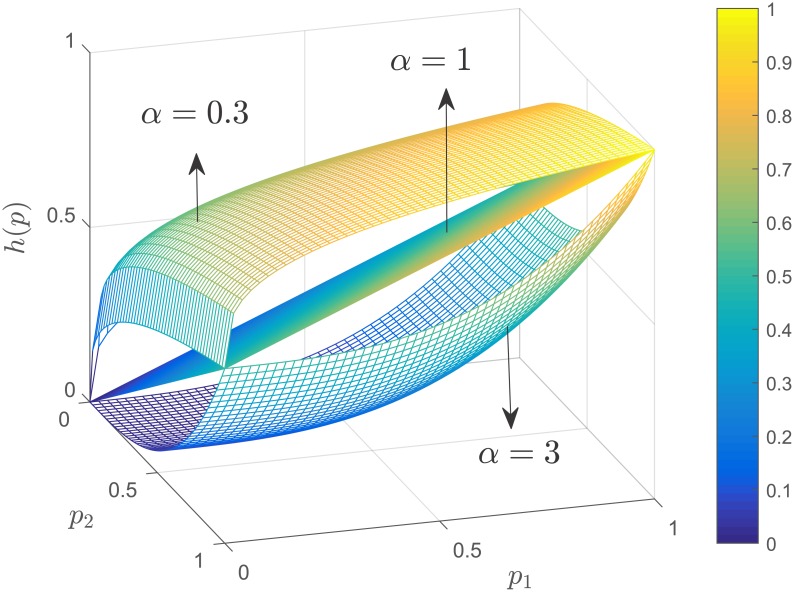
Production function in Eq (3) when *n* = 2, *α* = *α*_1_ = *α*_2_ = *α*_3_ ∈ {0.3, 1, 3} and *β* = *β*_1_ = *β*_2_ = *β*_3_ = 0.5. When *α* = 0.3, the production function exhibits a diminishing marginal productivity with respect to the participation load, opposite to the case when *α* = 3, which shows an increasing marginal productivity. A linear relation between contribution and participation is produced when *α* = 1.

In the context of the public goods problem, several types of production functions have been proposed to describe the relationship between the number of individuals cooperating in the production of the public goods and the amount of public goods that are produced ([12],Ch 4), [3, 28]. In the task completion problem, we use production functions with a structure that can be considered an extension of the ones presented in the problem of providing public goods, where we characterize the relationship between each individual’s participation load and their contribution to the completion of the task. A family of functions that captures different patterns in an individual’s contribution to the task is defined by

$$h\left(p\left(t\right)\right)=\sum_{i=1}^{n}\beta_{i}p_{i}^{\alpha_{i}}\left(t\right)$$ (3)

where *β*_*i*_ ≥ 0 and *α*_*i*_ > 0 are parameters associated with individual *i*. This family of functions describes the collective action of the community as the additive contribution of each individual’s participation load to the task completion. When *α*_*i*_ ∈ (0, 1), an individual exhibits a diminishing marginal productivity with respect to the participation load. The first few units of participation load have a significant impact on the individual’s contribution to the task. As the amount of participation load taken by the individual increases, his/her contribution has progressively less impact. In ([12],Ch 4), these types of functions are called *decelerating* production functions. On the other hand, when *α*_*i*_ > 1, small participation loads provide small contributions to the task completion. However, successive increases of the participation load provide progressively more significant contributions. In ([12],Ch 4), these types of functions are called *accelerating* functions. A linear relation between the individual’s contribution to task completion and his/her participation load is produced when *α*_*i*_ = 1. Fig 1 shows an example of these three cases.

#### Convergence Analysis

We provide some sufficient conditions on the parameters of the model that guarantee the completion of the task, and show through simulations the qualitative properties of the task convergence for different values of the parameters of the production function and participation load variables.

The next theorem states sufficient conditions on *ϕ* and *h* so that *z* converges to $\bar{z}$. The proof of this theorem is in S1 Appendix.

**Theorem 1.** *Let* $0\leq z\left(0\right)\leq \bar{z}$. *Assume that* *h*(*p*) ∈ [0, 1) *for all* *p* ∈ [0, *P*]^*n*^. *Also*, *assume that* *ϕh*(*p*(*t*)) *is such that*, *for all* *t* ≥ 0,

$$h\left(p\left(t\right)\right)\left|\bar{z}-z\left(t\right)\right|\geq \psi \left(\left|\bar{z}-z\left(t\right)\right|\right)$$ (4)

*where* *ψ* *is a strictly increasing function that satisfies* *ψ*(0) = 0. *Then*, *z*(*t*) *in* Eq (2) *satisfies* $0\leq z\left(t\right)\leq \bar{z}$ *for all* *t* ≥ 0 *and converges to* $\bar{z}$.

*Corollary* 1.1 Assume that *h*(*p*(*t*)) is fixed for all *t* ≥ 0. Then, *z* converges to $\bar{z}$ exponentially, and its solution trajectory is

$$z\left(t\right)=\bar{z}-{\left[1-\phi h(p(t))\right]}^{t}\left(\bar{z}-z\left(0\right)\right).$$ (5)

The assumption that *h*(*p*) ∈ [0, 1) does not make the formulation less general. Since the participation load *p*(*t*) is bounded for every *t* ≥ 0, the range of the production function is bounded below by zero and above by *P*, and therefore it is generally easy to formulate a production function that satisfies *h*(*p*(*t*)) ∈ [0, 1) for all *t* ≥ 0. In general, the assumption in Eq (4) implies that a task that is modeled using Eq (2) can be completed when the individuals behave such that *h*(*p*(*t*)) > 0 for all *t* ≥ 0. Later, we will use this result to to show that the participation strategy we propose guarantees the eventual completion of the task.

**Fig 2:**
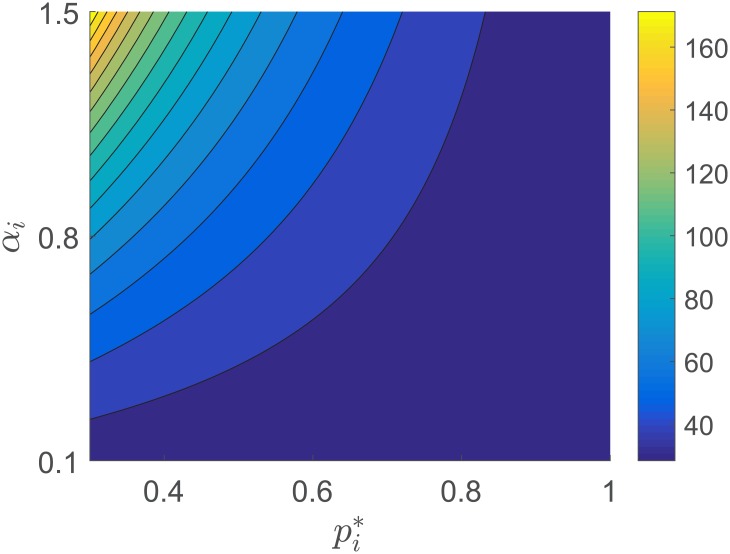
Contour plot of the convergence time t* in Eq (6) as a function of the production function parameter *α* and the participation load $p_{i}^{*}$, when only one individual participates in the task completion process. In this scenario, lower values of *α*_*i*_ are associated with smaller convergence times t*.

**Fig 3:**
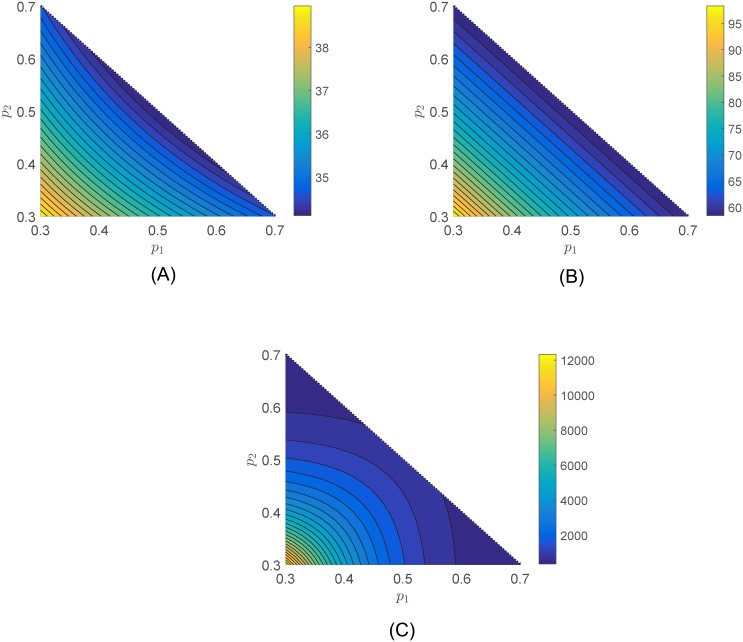
Contour plot of the convergence time t* in Eq (6) as a function of $p_{1}^{*}$ and $p_{2}^{*}$, where $p_{1}^{*}+p_{1}^{*}\leq 1$, when (A) *α* = 0.25, (B) *α* = 1, and (C) *α* = 5. When *α* = 1, the contribution of each individual is proportional to his/her participation load. When *α* = 0.25, the individuals can decrease their participation load and keep the same convergence time as long as both individuals share the participate load during the process of task completion. The opposite situation occurs when *α* = 5, where the convergence rate will increase if both individuals decide to participate and share the participation load.

From Corollary 1.1, we have that if the participation variables are assumed to be fixed for all *t*, the task completion variable will decrease exponentially with a convergence rate 1 − *ϕh*(*p*(*t*)). The task is completed when the expression ([1 − *ϕh*(*p*(*t*))]^*t*^ is zero. Next, we analyze the effect of the production function and the participation load on the convergence of the task. Assume that the individuals take a fixed participation load *p*(*t*) = p* ∈ [0, *P*]^*n*^ for all *t* ≥ 0, with $\sum_{i=1}^{n}\;p_{i}^{*}\leq P$, and let *σ* = [1 − *ϕh*(p*)]^t^*, where 1 − *σ* is the proportion of the task that has been already completed after t* iterations. Then, the minimum number of iterations taken to have a proportion 1 − *σ* of the task completed is given by

$$t^{*}=\left\lceil \frac{\text{log}\left(1-\sigma \right)}{\text{log}\left(1-\phi h\left(p^{*}\right)\right)}\right\rceil$$ (6)

where ⌈⋅⌉ denotes the ceiling function. As expected, the minimum number of time steps required to complete a proportion 1 − *σ* of the task decreases as the contribution provided by the individuals increases. Figs 2 and 3 show the minimum number of iterations t* needed to complete 95% of the task, i.e., *σ* = 0.05. We choose *ϕ* = 0.1, *P* = 1, and the production function in Eq (3) with *β*_*i*_ = 1/*n* for all *i* = 1, …, *n*. Fig 2 shows the contour plot of t* as a function of $p_{i}^{*}$ and *α*_*i*_ in Eq (3). Lower values of *α*_*i*_ are associated with smaller convergence times t*. For this specific production function, individuals provide a more significant contribution for low participation loads when *α*_*i*_ is smaller. Fig 3 shows the behavior of the convergence time t* as a function of $p_{1}^{*}$ and $p_{2}^{*}$ when *α* = *α*_1_ = *α*_2_ ∈ {0.25, 1, 5}, where $p_{1}^{*}+p_{2}^{*}\leq 1$. When *α* = 1, the contribution of each individual is proportional to his/her participation load. When *α* = 0.25, the individuals can decrease their participation load and keep the same convergence time as long as both individuals share the participate load during the process of task completion. The opposite situation occurs when *α* = 5. The convergence rate will increase if both individuals decide to participate and share the participation load.

### Motivation and Participation Costs

In the task completion problem, individuals obtain a benefit from the task once the task has been completed, and have to pay a cost proportional to their participation load if they want to contribute to the task completion. Therefore, an individual who decides to participate has to have the willingness to assume the participation costs associated with the task completion process. Hence, we define the *motivation* function *γ*_*i*_(*p*_*i*_, *t*) associated with the task as a function that quantifies the ability of individual *i*, at time step *t* and given his/her current participation load *p*_*i*_, to take additional participation load during the task completion process. There are different effects that have been observed that can cause loss or gain of motivation in individuals who are in a community. For example, *social loafing* is the effect when the individual’s motivation to participate has an inverse relationship with the size of the community [34, 35]. Also, *free riding* is the situation where an individual decides not to participate and expects others to get involved in the task completion process, and the *sucker effect* is the situation where an individual’s motivation decreases if other community members are free riding [34, 36]. On the other hand, the *Köhler effect* is an increase in motivation when an individual’s contribution to the task is lower than the other members of the community, or seen as indispensable to the completion of the task [42]. Other effects that motivate voluntary cooperative behaviors are discussed in [22]. Hence, the specific choice of the motivation function *γ*_*i*_(*t*, *p*_*i*_) depends on different phenomena, including the perception of the individual of the importance of completing the task, his/her ability to foresee the benefits after the task is completed, the number of people in the community, and his/her marginal productivity during the task completion process.

Let *c*_*i*_(*p*_*i*_) ≥ 0 be a function of *p*_*i*_ that quantifies the costs of participation for individual *i*. An expression that measures the willingness of the individual to take an additional unit of participation load is defined as the individual’s “marginal gain” function

$$\gamma_{i}\left(p_{i},t\right)-c_{i}\left(p_{i}\right).$$ (7)

The *social dilemma* arises when the motivation of the community members is less or equal than the costs associated with participation, and the collective efforts are not enough to complete the task. In this case, the marginal gain is non-positive, indicating that the willingness of the individuals to take additional units of participation load is not enough for them to engage in the participatory process. This state where no individual participates is called a *deficient equilibrium*. “It is deficient in that there is at least one other outcome in which everyone is better off, and it is an equilibrium in that no one has an incentive to change their behavior” ([3],p 184). In the next section we will provide conditions that allow the community to avoid this deficient equilibrium through a mechanism that enhances the motivation of an individual to take additional loads of participation in the process of task completion.

### Communication and Relative Reputation

We assume that the *n* individuals in the community are able to *communicate* across a network whose topology is represented by an undirected graph *G* = (*H*, *E*), where *H* = {1, …, *n*} is the set of nodes and *E* ⊂ *H* × *H* is the set of edges. Each node is associated with an individual, and each edge is associated with the interaction between two individuals. Edge (*i*, *j*) indicates that individuals *i* and *j* share information about their participation and marginal gain. The individuals who interact with individual *i* are his/her neighbors *N*_*i*_ = {*j* ∈ *H*: (*i*, *j*) ∈ *E*}.

According to Eq (7), the marginal gain to an individual of the participatory action depends on the individual’s motivation to complete the task. An individual with motivation less than or equal to the participation costs will not be willing take additional units of participation load. A mechanism that is known that can help promote participation in the community is *indirect reciprocity* [41]; an individual’s motivation can increase if his/her neighbors also participate in the task completion process. In order to model this mechanism, we first introduce the concept of *relative reputation*. Let *j* ∈ *N*_*i*_. We define the relative reputation of individual *j* from individual *i*’s viewpoint as the individual *i*’s recognition of individual *j*’s involvement in completing the task. The process of *j* building a reputation from *i*’s viewpoint can be characterized using the update rule

$$r_{ij}\left(t+1\right)=\left(1-\tau_{ij}\right)r_{ij}\left(t\right)+\tau_{ij}\frac{p_{j}\left(t\right)}{P}\bar{r}$$ (8)

where *τ*_*ij*_ ∈ [0, 1] is the rate of change in reputation, and $\bar{r}\geq 0$ is the upper bound of reputation. A value of *τ*_*ij*_ close to one implies that the reputation is highly influenced by the participation load, and develops according to it. Assuming that $r_{ij}\left(0\right)\in \left[0,\bar{r}\right]$, *τ*_*ij*_ > 0, and that the participation load $p_{j}\left(t\right)=p_{j}^{*}$ is fixed for all *t* ≥ 0, we have that

$$\lim_{t\to \infty }r_{ij}\left(t\right)=\bar{r}\frac{p_{j}^{*}}{P}.$$

When *τ*_*ij*_ = 1, the relative reputation reaches its maximum value $\bar{r}p_{j}^{*}/P$ at one iteration. On the other hand, when *τ*_*ij*_ = 0, the relative reputation does not change and remains at its initial value *r*_*ij*_(0). The term (1 − *τ*_*ij*_)*r*_*ij*_(*t*) in Eq (8) can be seen as a “forgetting factor:” if the current participation load taken by individual *j* is greater than zero, individual *i* forgets past participation patterns and adjusts *j*’s reputation accordingly. If the participation load taken by individual *j* is zero, then his/her reputation with respect to *i*’ viewpoint will decrease and reach zero asymptotically.

The *reciprocity* of individual *i* to participate in the task is then quantified as the linear combination of the reputation of his/her neighbors, i.e., for *i* = 1, …, *n*,

$$r_{i}\left(t\right)=\sum_{j\in N_{i}}\delta_{ij}r_{ij}\left(t\right),$$ (9)

with *δ*_*ij*_ ∈ [0, 1]. If *δ*_*ij*_ = 0 (> 0), then *i* does not (does, respectively) consider *j* for reciprocal participatory actions. Reciprocity acts as a catalyst for participation. An individual’s motivation to participate can be enhanced by his/her reciprocity to his/her neighbors’ actions. The marginal gain at time *t* to an individual that has a participation load *p*_*i*_(*t*) of the task completion action in Eq (7) can be generalized to include reciprocity as

$$g_{i}\left(p_{i}\left(t\right),t\right)=b_{i}\left(p_{i}\left(t\right),t\right)-c_{i}\left(p_{i}\left(t\right)\right)$$ (10)

where

$$b_{i}\left(p_{i}\left(t\right),t\right)=\gamma_{i}\left(p_{i}\left(t\right),t\right)+r_{i}\left(t\right).$$

If an individual’s motivation through reciprocity is strong enough, i.e., *r*_*i*_(*t*) > *c*_*i*_(*p*_*i*_(*t*)) − *γ*_*i*_(*p*_*i*_(*t*), *t*), then the individual will engage in the participatory process. In this case, if at least one individual is willing to participate, the interaction network is connected (i.e., there is a path between every two nodes in the network), and the parameters associated with reciprocity allow its development, then the members of the community will eventually be willing to participate in the task completion process, and come out the deficient equilibrium.

Recall that the community of individuals can take a total participation load up to *P*. Next, we will show how communication also allows the individuals to collaborate by locally distributing the available participation load *P* between them based on their individual motivation and participation, and the interaction patterns in the network.

## Collective Action

A strategy that promotes cooperation is one where, based on local interactions, the individuals seek to distribute the available participation load such that the amount of participation is maximized based on the individuals’ marginal gain, and the constraint in Eq (1) is satisfied. Cooperative individuals with higher motivation and reciprocity will be willing to assume higher costs of participation, which implies taking larger participation loads than those with lower motivation and reciprocity. In this way, a community whose members *cooperate* can be seen as an economic agent with unlimited wants (participation load taken by members as large as possible) with limited resources (available task participation load *P*). From this point of view, an individual’s marginal gain is the additional “satisfaction” that the community gains from assigning one more unit of participation load to that individual. The community then distributes its limited available task participation load among various members to increase its level of satisfaction, where the marginal satisfaction of each member corresponds to the individual’s marginal gain in Eq (10) as a function of the participation load. Hence, assuming that *g*_*i*_ is a function of only *p*_*i*_, the level of satisfaction of the community can be expressed as

$$U\left(p\right)=\sum_{i=1}^{n}u_{i}\left(p_{i}\right)$$ (11)

where

$$u_{i}\left(p_{i}\right)=\int_{0}^{p_{i}}g_{i}\left({\tilde{p}}_{i}\right)\;d{\tilde{p}}_{i}=B\left(p_{i}\right)-C\left(p_{i}\right),$$

and

$$B\left(p_{i}\right)=\int_{0}^{p_{i}}b_{i}\left({\tilde{p}}_{i}\right)\;d{\tilde{p}}_{i},\;\;\;C\left(p_{i}\right)=\int_{0}^{p_{i}}c_{i}\left({\tilde{p}}_{i}\right)\;d{\tilde{p}}_{i}$$

Individuals with larger (lower) marginal gains will take larger (lower, respectively) participation loads in order to maximize the community’s level of satisfaction *U*. Note that if we assume that *b*_*i*_ = ∂*h*/∂*p*_*i*_, then *u*_*i*_ = *B*(*p*_*i*_) − *C*(*p*_*i*_) has the same structure as the net gain presented in the context of collective good models in ([12],Eq 1).

In the formulation in Eq (11), we are assuming that the marginal gain is a function of *p*_*i*_. However, it can be the case that the parameters of the marginal gain change over time, changing the structure of the maximization problem. In this section, we study how cooperative individuals act at each time step given a specific structure of optimization problem.

The distribution of the available task participation load among the community members becomes a maximization problem, where the objective function is the level of satisfaction in Eq (11) subject to the constraint in Eq (1). In order to have a well-defined optimization problem, we make some assumptions on the values that each *p*_*i*_ can take and on the marginal gain as a function of *p*. First, we assume that the marginal gain *g*_*i*_ is monotonically decreasing with respect to *p*_*i*_ ∈ [0, *P*]. This assumption implies that an individual’s willingness to take more units of participation load decreases as his/her participation load increases, guaranteeing that *U*(*p*) is a concave function of *p*. Also, we assume that *g*_*i*_ is positive for *p*_*i*_ ∈ [0, *P*]. This indicates that an individual that wants to cooperate, has to have a minimum willingness to assume the costs associated with the total participation load *P*. Hence, we redefine the marginal gain as

$$g_{i}\left(p_{i}\left(t\right)\right)=\left\{\begin{array}{cc} b_{i}\left(p_{i}\left(t\right)\right)-c_{i}\left(p_{i}\left(t\right)\right), & \text{if}\;b_{i}\left(P\right)>c_{i}\left(P\right)\\ 0, & \text{otherwise} \end{array}\right.$$

There is a large variety of families of functions that satisfy these conditions and can capture different participation dynamics in the community. For example, if the individual’s motivation *b*_*i*_ is fixed, and the costs of participation are assumed to be linear with the form *c*_*i*_*p*_*i*_, then an individual who cooperates will have a motivation that satisfies *b*_*i*_/*c*_*i*_ > *P*. This function describes an individual whose willingness to take an additional unit of participation load decreases as *p*_*i*_ increases. On the other hand, if the individuals in the community do not consider the participation costs associated with the completion of the task, and their motivation is based on their marginal productivity ∂*h*/∂*p*_*i*_, the community’s level of satisfaction in Eq (11) will be the same production function (assuming that the production function is concave). In this case, the individuals will allocate the available participation load so that their combined productivity to complete the task is maximized.

Using these assumptions, the next theorem provides some properties of the solution of the optimization problem that will be useful in the formulation of an algorithm for the decentralized passing (distribution) of the participation load across the network.

**Theorem 2.** *Let* *n* ≥ 2. *Consider the optimization problem*

$$\underset{p\in R^{n}}{maximize}\;\;\;U\left(p\right)$$

$$\begin{array}{ll} subject\;to & \sum_{i=1}^{n}p_{i}\;\leq \;P\\ & 0\;\leq \;p_{i},\;i\;=\;1,\;\ldots ,\;n \end{array}$$

*Let* $p^{*}\in R_{\geq 0}^{n}$, μ* > 0, *and* $\lambda_{i}^{*}\geq 0$, *i* = 1, …, *n* *satisfy*

$$\sum_{i=1}^{n}p_{i}^{*}=P$$ (12)

*and*

$$g_{i}\left(p_{i}^{*}\right)=\mu^{*}-\lambda_{i}^{*}>0,\;\;\;i=1,\ldots ,n$$ (13)

*where* $\lambda_{i}^{*}=0$ *if* $p_{i}^{*}>0$. *Then*, p* *is a strict maximum of* *U* *over the feasible region*.

*Corollary* 2.1 If p* is a strict maximum of *U* over the feasible region, then p* is the only point that satisfies

$$\sum_{i=1}^{n}p_{i}^{*}=P$$

and

$$g_{i}\left(p_{i}^{*}\right)=g_{i}\left(p_{j}^{*}\right)$$

for all $p_{i}^{*},p_{j}^{*}>0$, *i* ≠ *j*, *i*, *j* = 1, …, *n*.

Corollary 2.1 states that, under the conditions on the values that *p* can take and on the marginal gain as a function of *p*, cooperation in the community involves a process where the individuals tend to equalize their marginal gain. Individuals with larger marginal gains will take more participation load than individuals with lower marginal gain. It can be the case that some individuals do not take any participation load to allow others who consistently have larger marginal gains to get all the available participation load.

Based on this result, we can study the effect of the interaction patterns in the community on the dynamics of cooperation by designing a *cooperation* strategy that *locally* distributes the available participation load. Here, each individual only knows the information from his/her neighbors in the network. The community acts in a way that the individuals share the participation load and seek to balance their individual marginal gain between them during the process.

We define the dynamics of participation following the algorithm presented in [31], where each individual can take (pass) an amount of load from (to) some of his/her neighbors based on their marginal gain. These dynamics are characterized by

$$p_{i}\left(t+1\right)=p_{i}\left(t\right)-\sum_{j\in S_{i}}L_{j}^{i}\left(t\right)+\sum_{\left\{l:i\in N_{l}\right\}}L_{i}^{l}\left(t\right)$$ (14)

where $L_{j}^{i}\left(t\right)$ denotes the participation load that individual *i* is passing to *j*, and $L_{i}^{l}\left(t\right)$ denotes the participation load that *i* receives from individual *l*, with *i* ∈ *N*_*l*_. We formulate the participation dynamics in Eq (14) so that the individuals tend to equalize their individual marginal gain based on information from their neighbors, and the constraint $\sum_{i=1}^{n}\;p_{i}=P$ is satisfied.

A cooperation policy that has these characteristics can be summarized by the following rules:

1. An individual that has neighbors with larger gains will pass some amount of participation load to them, since they are more willing to participate in the task ($L_{j}^{i}\left(t\right)$ in Eq (14)).
2. After *i* passes an amount of participation load to *j*, the marginal gain of *j* cannot be lower than the marginal gain of *i*.
3. An individual cannot pass an amount of participation load that is greater than the one that he/she currently has.

Rule 1 is based on the fact that *g*_*i*_ is a decreasing function of *p*_*i*_. It defines who individual *i* should pass an amount of participation load to. Rules 2 and 3 constrain the amount of participation that an individual can pass to his/her neighbors.

To provide a mathematical formulation of these rules, we first define the set *S*_*i*_:

$$\begin{array}{ll} S_{i}= & \{j\in N_{i}:g_{j}\left(p_{j}\left(t\right)\right)\;>\;g_{i}\left(p_{i}\left(t\right)\right),\\ & g_{j}\left(p_{j}\left(t\right)\right)\geq g_{l}\left(p_{l}\left(t\right)\right),\;\forall l\in N_{i}\} \end{array}$$ (15)

This set contains individual *i*’s neighbors with marginal gain larger than *i*’s marginal gain and have the largest marginal gain among *i*’s neighbors at time step *t*. It identifies those individuals who need to take a participation load from *i* so that their marginal gain can be equalized with *i*’s marginal gain. Below, we give the algorithm with the mathematical description of the steps that the individuals follow to compute the participation loads to be passed at each time.

**Algorithm: Distribution of participation load**. Let *p*(*t*) satisfy $\sum_{i=1}^{P}\;p_{i}\left(t\right)=P$, *p*_*i*_(*t*) ≥ 0, and *g*_*i*_(*p*_*i*_(*t*)) > 0 for all *p*_*i*_ ∈ [0, *P*], *i* = 1, …, *n*. Given the interaction network *G* = (*H*, *E*), the current participation load *p*(*t*), the load passing rate *θ*, and the individuals’ marginal gain, the algorithm to compute $L_{j}^{i}\left(t\right)$ and $L_{i}^{l}\left(t\right)$ in Eq (14) is:

1: **Input:** *G*, *p*(*t*), *θ* ∈ (0, 1], and *g*_*i*_ for all *i* ∈ *H*.

2: **Output:** $L_{j}^{i}\left(t\right)$ for all *i* ∈ *H* and *j* ∈ *N*_*i*_.

3: **for** each *i* ∈ *H* **do**

4: Choose the $L_{j}^{i}\left(t\right)$ so that:

5:  $L_{j}^{i}\left(t\right)=0$ if $j\notin S_{i}$, and

6:  $\sum_{j\in N_{i}}L_{j}^{i}\leq p_{i}$, and

7:  $g_{i}\left(p_{i}-\sum_{i=1}^{n}L_{j}^{i}\right)\leq g_{j}\left(p_{j}+L_{j}^{i}\right)$, and

8:  $g_{j}\left(p_{j}+L_{j}^{i}\right)\leq g_{j}\left(p_{j}\right)-\theta (g_{j}\left(p_{j}\right)-g_{i}\left(p_{i}\right))$

9: **end for**

This algorithm is formulated based on the assumption that the marginal gain *g*_*i*_ is decreasing with respect to *p*_*i*_. If $S_{i}$ in Eq (15) is not empty, then it means that individual *i* can share participation load with a neighbor and try to equalize their marginal gain. This action is stated in line 8 in the algorithm (rule 1). Lines 7 states that an individual cannot pass more than his/her current participation load. Line 9 guarantees that the marginal gains will be equalized at an exponential rate as we will show in the next theorem.

Note that, in the distribution algorithm, each individual only knows the current marginal gain and participation load of his/her neighbors. Only individuals whose marginal gain is *g*_*i*_(*p*_*i*_) > 0 for all *p*_*i*_[0, *P*] are considered in the participation process. The next theorem states that this decentralized distribution algorithm, along with Eq (14), leads to a distribution of the participation load where the marginal gains of the individuals are equalized.

**Theorem 3.** *Assume that* *g*_*i*_(*p*_*i*_) > 0 *for* *p*_*i*_ ∈ [0, *P*], *i* = 1, …, *n*. *Also*, *assume that the optimization algorithm in Theorem 2 has a maximizer with positive entries*. *Let* *p*_*i*_(0) ≥ 0 *satisfy* $\sum_{i=1}^{n}\;p_{i}\left(0\right)=P$, *and let the network be connected*. *Then*, *the iterative computation of the algorithm along with* Eq (14) *will lead to a distribution of the participation load where the state* *g*_*i*_(*p*_*i*_) = *g*_*j*_(*p*_*j*_), *for every pair of nodes in the network*, *is invariant and is reached at an exponential rate*.

This theorem is a straightforward result of ([31],Theorem 3.4). It states that if there is a path between every two nodes in the network, then it can be guaranteed that the individuals that have the ability to communicate will equalize their marginal gains. This implies that those individuals that are more (less) willing to participate will eventually take more (less, respectively) participation load in the task completion process.

**Fig 4:**
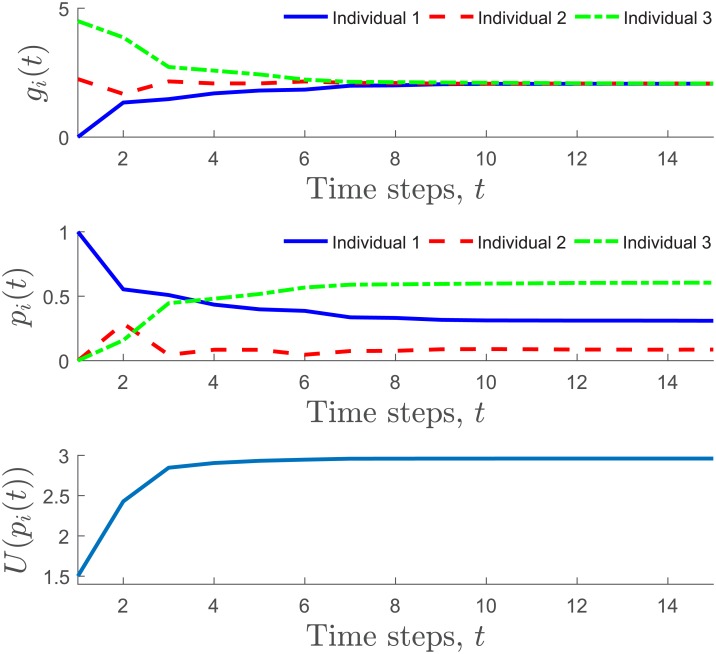
Example of the marginal gain balancing between three individuals that interact across a line topology. The marginal gain (top plot), the participation load (middle plot) for each one of the individuals, and the utility function in Eq (11), are shown throughout the iterative process in Eq (14) and the load distribution algorithm.The participation loads are dynamically distributed while the utility function is maximized. The Matlab code is in S1 Appendix.

**Fig 5:**
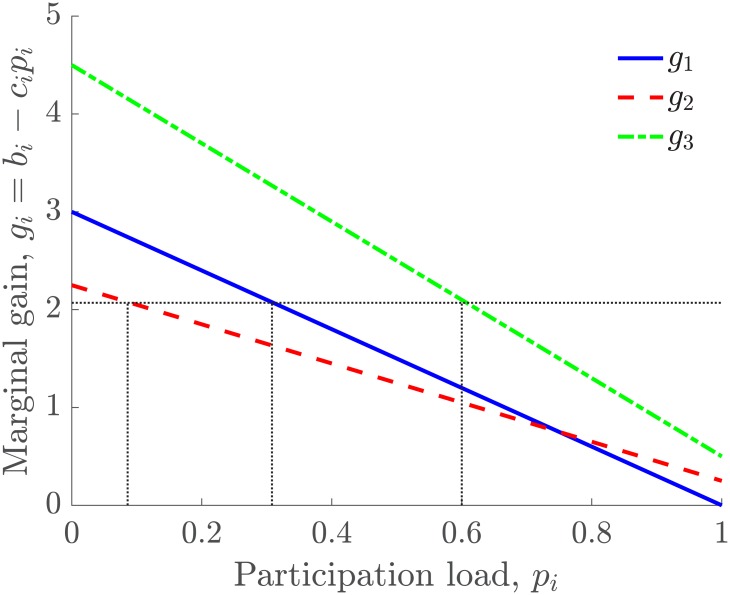
Marginal gain *g*_*i*_ = *b*_*i*_ − *c*_*i*_*p*_*i*_ as a function of *p*_*i*_, for *i* = 1, 2, 3 at time *t*. The dotted lines indicate the distributed participation loads $p_{i}^{*}$, and the equalized marginal gains g* that result from the iterative process shown in Fig 4.

Fig 4 shows an example of the trajectories of *p*_*i*_ and *g*_*i*_ throughout the iterative process. The three individuals interact according to a network with a line topology (one individual with two neighbors, the other two individuals have one neighbor). The total participation load is *P* = 1, and the load passing load is *θ* = 0.005. The benefits are set to be *b*_1_ = 3, *b*_2_ = 2.25, and *b*_3_ = 4.5, and the participation costs are *c*_1_ = 3, *c*_2_ = 2, and *c*_3_ = 4. As they interact locally according to the network and pass the participation load, the marginal gain for all the individuals tends to equalize during the process. Note that the participation loads are dynamically distributed so that $\sum_{i=3}^{n}\;p_{i}\left(t\right)=P$ for all *t* ≥ 0 and they are proportional to the individual motivation. The level of satisfaction of the community given in Eq (11) is maximized during the process. Fig 5 shows the marginal gain *g*_*i*_ as a function of *p*_*i*_, for *i* = 1, 2, 3. The dotted lines indicate the location of the distributed participation loads and the equalized marginal gains. The algorithm found the participation load distribution so that *g*_1_(*p*_1_) = *g*_2_(*p*_2_) = *g*_3_(*p*_3_) and $\sum_{i=1}^{3}\;p_{i}\leq P$.

Next, we study the conditions that guarantee the completion of tasks when the individuals distribute their participation load following the algorithm above. We will show in Theorem 4 sufficient conditions on *b*_*i*_ so that the convergence of *z*(*t*) to $\bar{z}$ is guaranteed.

**Theorem 4.** *Let* $\sum_{i=1}^{n}\;p_{i}\left(0\right)=0$, *and let the production function be such that* *h*(*p*) ∈ [0, 1) *for all* *p* ∈ [0, *P*]^*n*^. *Assume that*, *at each iteration step* *t*, *the individuals distribute their participation load following* Eq (14) *and the algorithm for participation load distribution*. *Assume that*, *at each time* *t* ≥ 0, *at least one individual* *i* ∈ *H* *has motivation and reciprocity* *b*_*i*_(*p*_*i*_, *t*) *so that* *g*_*i*_(*p*_*i*_, *t*) > 0 *for* *p*_*i*_ ∈ [0, *P*]. *Then*, *z*(*t*) *in* Eq (2) *will converge to* $\bar{z}$.

The conditions on the motivation and reciprocity presented in this theorem are a consequence of the result in Theorem 1. If at each time step there is at least one individual that has the willingness to participate, then the task will eventually be completed. This theorem only provides information about the basic conditions so that the completion of the task is guaranteed. Next, we will study the effect of the production function, topology of the network, and size of the community on the participation dynamics.

## Analysis of Participation Dynamics

To study the dynamics that result from the interaction between the load distribution algorithm, reciprocity, production function, and topology of the network, we assume that the individuals’ motivation is constant during the iterative process, and the costs of participation are linear with respect to the participation load. We study the dynamics of the task completion problem in two simulation scenarios. First, we take a scenario with five individuals and a specific selection of the model parameters and topology of the interaction network. We show how the participation, task completion, marginal gain, and relative reputation variables change over time. Second, using Monte Carlo simulations, we show the behavior of the community for different production functions, size of the community, and topologies when some of the model parameters are randomly sampled.

### Participation Patterns in the Community

**Fig 6:**
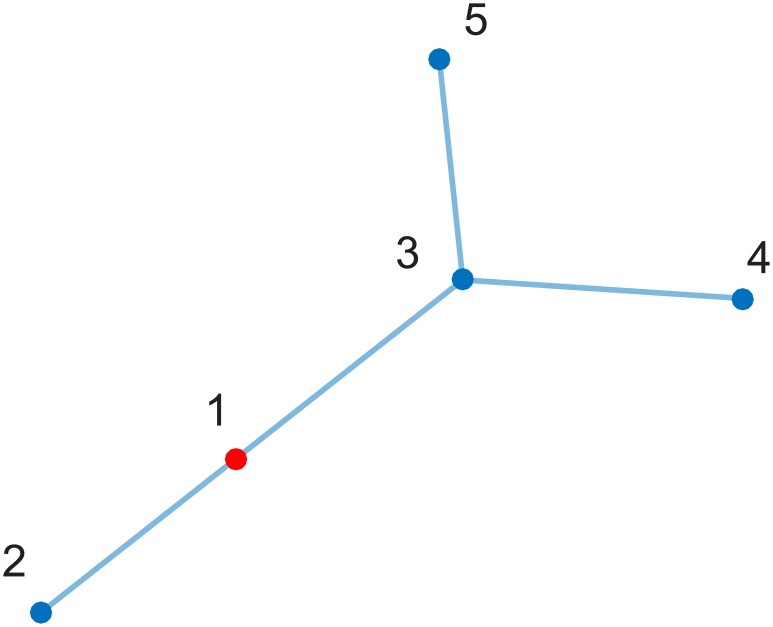
Topology of the interaction network, where each node is associated with an individual, and each link denotes interaction between two individuals.

We illustrate the evolution of the variables involved in the task completion dynamics for a specific example. The community has *n* = 5 individuals who communicate following the network shown in Fig 6. In this example, individuals have a marginal gain defined as *g*_*i*_(*t*) = *γ*_*i*_ + *r*_*i*_(*t*) − *c*_*i*_*p*(*t*), where *c*_*i*_ > 0 is fixed, *γ*_1_ > 0, *γ*_*i*_ = 0 for *i* = 2, …, *n*, and *r*_*i*_(0) = 0 for *i* = 1, …, *n*. During the distribution process, the marginal gain of those individuals with (*γ*_*i*_ + *r*_*i*_(*t*))/*c*_*i*_ < *P* is set to zero in order to ensure that only the individuals with marginal gain *g*_*i*_(*p*_*i*_) > 0 for *p*_*i*_ ∈ [0, *P*] are taken into account. The total participation load was *P* = 1. We assumed that all the neighbors contribute equally to the reciprocity of each individual (see Eq 9). The Matlab code to generate this simulation is provided in S1 Appendix.

**Fig 7:**
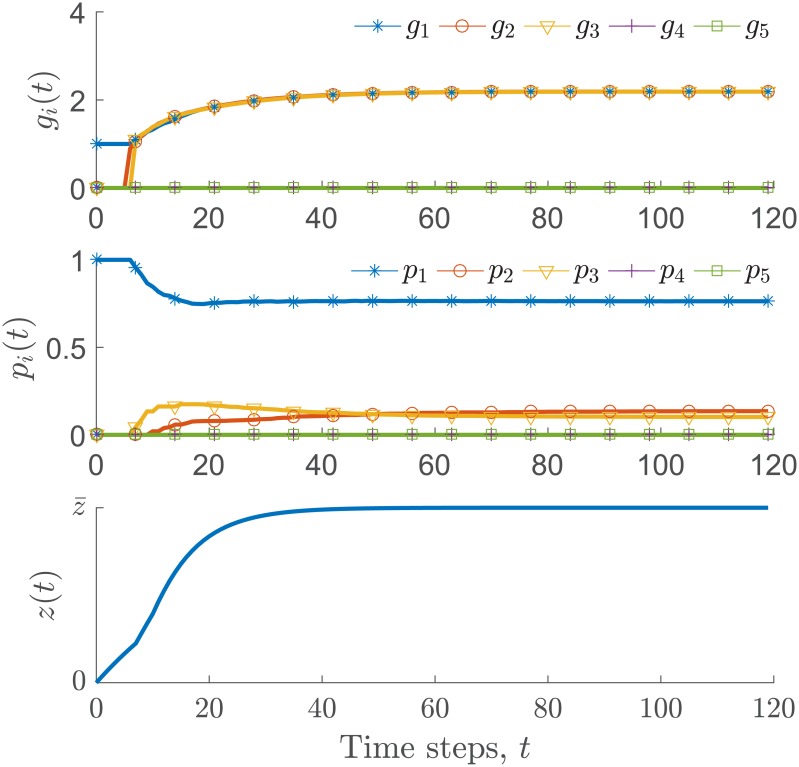
(Trajectories of the individuals’ participation load variables *p*_*i*_(*t*), marginal gain *g*_*i*_(*t*), and the task completion variable throughout the iterative process. During the first iterations of the participatory process, individual 1 increases his/her participation load, taking almost all the available load *P*. Due to the effect of reciprocity, individuals 2 and 3 increase their participation load at later iterations. Individuals 4 and 5 take a participation load close to zero because the relative reputation of individual 3 is not large enough to produce reciprocal actions in the participatory process.

Fig 7 shows that during the first iterations of the participatory process, individual 1 increases his/her participation load, taking almost all the available load *P*. Since his/her initiative *γ*_*i*_ is different from zero, individual 1 is the only member of the community who is initially willing to participate in the completion of the task. Since individuals 2 and 3 interact with 1, their reciprocity toward participation develops, as well as their participation load. Once their marginal gain is large enough to be considered in the process, their participation load increases accordingly. Note that the task completion growth rate increases when individuals 2 and 3 start participating (at around *t* = 7). Individuals 4 and 5 take a participation load close to zero because the relative reputation of individual 3 is not large enough to produce reciprocal actions in the participatory process.

**Fig 8:**
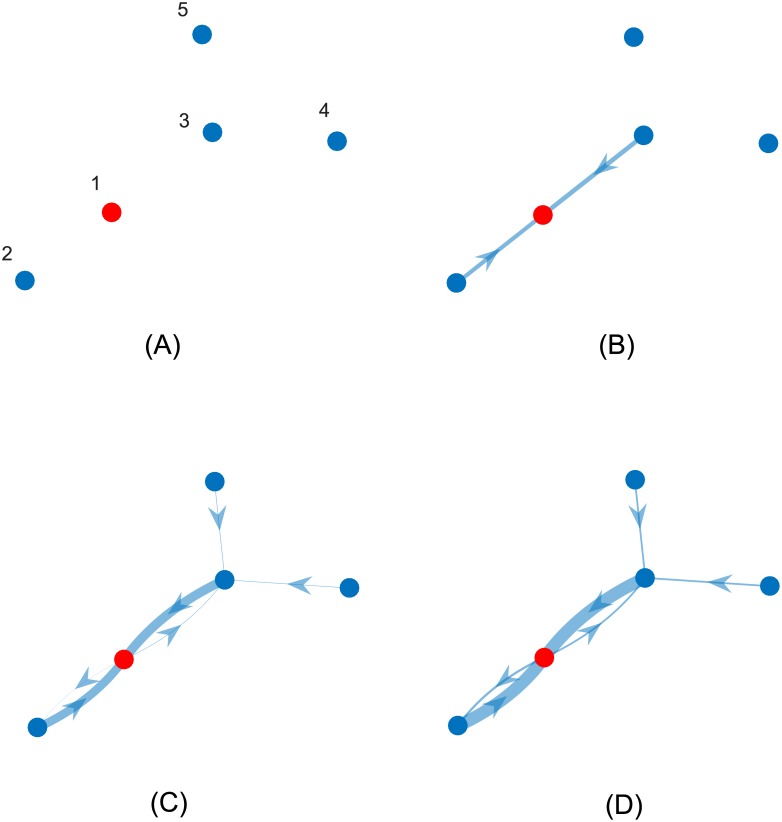
Evolution of the relative reputation *r*_*ij*_(*t*) in Eq (8) for time steps (A) *t* = 0, (B) *t* = 5, (C) *t* = 12, and (D) t = 100. Each node is associated with an individual, and each edge (*i*, *j*) (arrow going from *i* to *j*) is associated with *r*_*ij*_(*t*). The thickness of the edges is proportional to *r*_*ij*_(*t*). Only individual 1 has motivation *γ*_1_ grater than zero.

Fig 8 shows the evolution of the relative reciprocity of the individuals during the task completion process. The relative reputation *r*_*ij*_(*t*) in Eq (8) (reputation of *j* from *i*’s point of view) is represented as the edge of a graph that connects nodes *i* and *j* at time step *t* in the direction *j* → *i*, and whose thickness is proportional to *r*_*ij*_(*t*). At time *t* = 0 none of the individuals has developed any reputation (Fig 8A). Note that individual 1 builds his/her reputation from the viewpoint of individuals 2 and 3 since it has an early involvement in the task completion process (Fig 8B). Individuals 2 and 3 develop their reputation from their neighbor’s point of view as they take an amount of participation load (Fig 8C and 8D). Individuals 3, 4, and 5 do not build enough reputation during the process.

This set of simulations shows the effect of reputation on the dynamics of participation during the task completion process. Although only one individual has the motivation to assume the costs of participation in the task completion process, reciprocity through the relative reputation helps to promote participation. The effect of reciprocity is not immediate, since building the relative reputation is a dynamical process [41]. The load distribution algorithm allows the individuals to interact and distribute the available participation load according to the potential benefit that they take into consideration and their participation costs. As reciprocity increases, the distribution of the participation load changes as more individuals are willing to participate. Since the network in the example is connected, the motivation of individual 1 (who is the only individual with initiative different from zero) encourages reciprocal participation across the entire network following a “chain reaction.”

### Monte Carlo Simulations

In this scenario, for each simulation run only one individual in the community is randomly chosen to have a constant motivation *γ*_*i*_ which is different from zero. Also, the cost of participation *c*_*i*_ and the rate of increase in reputation *τ*_*ij*_ are randomly chosen from a uniform distribution. We assumed that the parameters *α*_*i*_ and *β*_*i*_ in the production function in Eq (3) were the same for all the individuals, and that the neighbors contributed equally to the computation of reciprocity in Eq (9). We conducted 6000 Monte Carlo runs in total, ensuring that the estimated median, and 25% and 75% percentiles converged. We explored situations with different production functions, topologies of the interaction network, and size of the community. The implementation details are in S1 Appendix.

#### Effect of the Production Function and Network Topology

**Fig 9:**
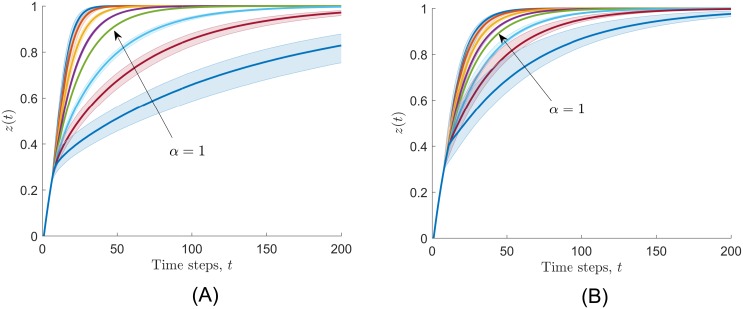
Evolution of the median (line) and 25% and 75% percentiles (lower and upper boundaries of the shaded region) of the task variable *z*(*t*) estimated from 6000 Monte Carlo runs for a (A) fully connected and (B) line topologies of the interaction network. The parameter of the production function *α* in Eq (3) takes the values 0.1, 0.25, 0.5, 0.75, 1, 1.5, 2, and 3, from the left (*α* = 0.1) to the right (*α* = 3) lines, respectively. In this scenario, individuals took longer to complete the task as *α* increased.

**Fig 10:**
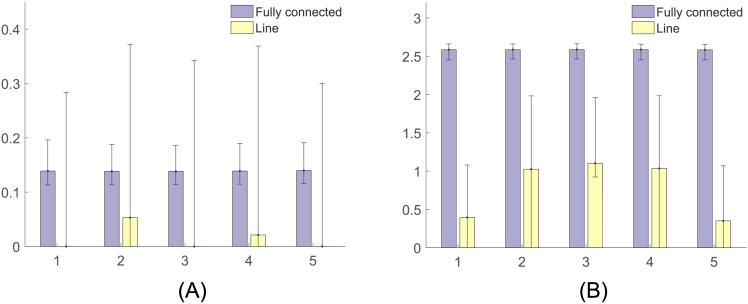
Median (bar plots) and 25% and 75% percentiles (error bars) of (A) the total participation load and (B) reciprocity per individual during the task completion process, estimated from 6000 Monte Carlo runs for a fully connected and line topologies of the interaction network. In communities with the fully connected network, individuals tend to achieve similar reciprocity and equal distribution of the participation load, while communities with the line topology tend to have lower reciprocity and unequal distributions of the participation load.

In the first set of simulations we tested the behavior of the individuals when they followed a fully connected and a line topology, for different values of the parameter *α* = *α*_*i*_, *i* = 1, …, *n*, in the production function in Eq (3). Recall that *α* determines the shape of the production function, and therefore the relationship between the participation load taken by the individuals and their contribution to the completion of the task. Fig 9 shows the median and the 25% and 75% percentiles of the task completion variable *z*(*t*) estimated from the Monte Carlo runs. Note that, for both network topologies, the individuals took longer to complete the task as *α* increased. However, for decelerating production functions (*α* < 1), communities following a fully connected topology had shorter convergence times than communities following a line topology. For accelerating production functions (*α* > 1), communities following a line topology had shorter convergence times than those following fully connected topologies. These convergence patterns in the task completion process arise due to the way that the total participation is distributed between the individuals. Fig 10A shows the participation patterns per individual for the last 100 time steps of the task completion process. In the fully connected case, individuals tend to distribute equally the participation load. Once the only individual that is motivated to participate is involved in the process, his/her relative reputation develops to the point where the rest of the community engages in the participatory process. As other people participate, their reputation increases, producing an increase in the other individuals’ reciprocity to participate. This enhanced willingness to participate in all the community leads to an equal distribution of the participation load. On the other hand, in the line case there is a tendency of large unequal distributions of the participation loads. Since at the beginning of the process only one individual is willing to participate, and each individual has at most two neighbors, reciprocity is no strong enough to involve every community member in the participatory process.

As it was shown in Fig 3, equal distributions of the participation load favor the productivity of the group in tasks with decelerating production functions, since individuals can be very productive with small loads of participation. On the other hand, unequal distributions favor the productivity of the community in tasks with accelerating production functions. It is better if one individual takes larger loads of participation, than distributing it between several community members.

Fig 10B shows the median and the 25% and 75% percentiles of the reciprocity variable per individual in Eq (9) for the last 100 time steps of the task completion process. In communities with the fully connected network, individuals tend to achieve similar reciprocity, since all community members are connected to each other. The reciprocity in communities with the line topology tends to be lower than in communities with the fully connected topology. Also, since individuals 1 and 5 only have one neighbor, the reciprocity that they develop is smaller compared to the reciprocity of individuals 2, 3, and 4.

#### Effect of the Number of Individuals and Network Topology

**Fig 11:**
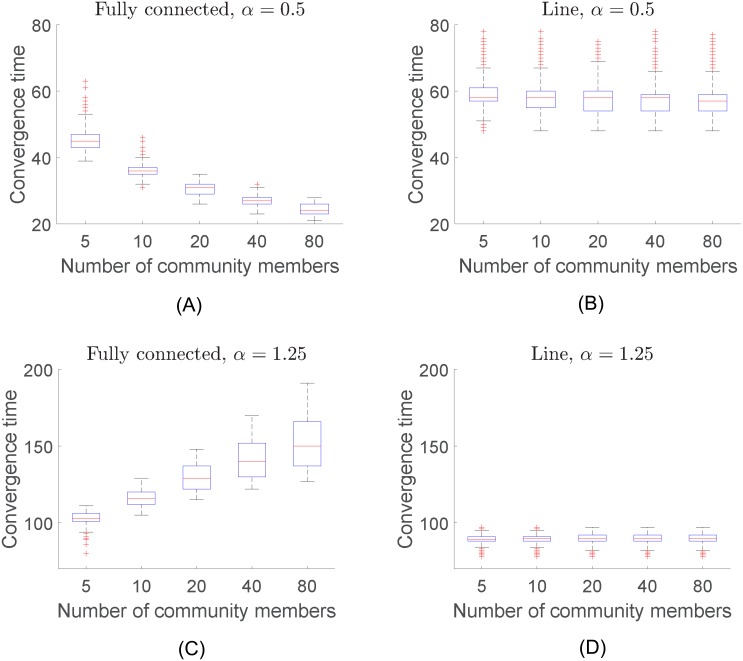
Box plots of the convergence time for (a) the parameter of the production function *α* = 0.5 and a fully connected network, (b) *α* = 0.5 and a line network, (c) *α* = 1.25 and a fully connected network, and (d) *α* = 1.25 and a line network. The size of the community has a significant effect on the convergence time in the case of the fully connected network topology. The opposite situation occurs in the case of the line topology.

In the second set of simulations, we studied the behavior of communities that varied in size. The total participation load *P* and the production function remained the same in all the simulations. We define the convergence time as the time step where the task variable *z*(*t*) first reaches 95% of its completion state $\bar{z}$. Fig 11 shows the behavior of the convergence time versus the number of individuals in the community after 6000 Monte Carlo runs for different combinations of the topology of the interaction network and the parameter *α* of the production function. These results show that, as the number of members increases, communities with a fully connected network tend to decrease the convergence time of the task completion for decelerating production functions (*α* < 1). In this case, the topology of the network favors participation of most of the members of the community, and therefore, due to the nature of the production function, providing an increase in their combined productivity. A different situation occurs in the case of accelerating functions (*α* > 1), where the productivity of the community decreases as more individuals are willing to participate in the task completion process.

In the case of the line topology, the size of the community has little effect on the convergence time of the completion of the task. The results are consistent with the results in Section: the convergence time in the line topology tends to be larger (shorter) for decelerating (accelerating, respectively) functions than the ones with the fully connected network.

## Metaphorical Use of the Model

This model provides a framework for understanding the conditions that promote participation of community members on a common task, and the different participation patterns that can result from collective action. The complexity of collective action in real settings makes impossible the incorporation all the elements that are involved in this process in a single model. However, this simplified model, used as a metaphor, provides insights about the community functioning for different elements that have been found to be key in the dynamics of collective action and cooperation, such as social motivation [22, 34], network interaction ([43],Ch 3), relative reputation and reciprocity ([44],Ch 2), and production function ([12],Ch 4).

An approach for community change that can benefit from the metaphorical use of our model is participatory action research (PAR). PAR provides some basic principles for involving people in participation and taking action to improve the community conditions [45],([26],Ch 5). Our model can serve as a tool to understand issues in the community for possible action, and identify strategies to address such issues. First, the production function is used in the model to represent how influential the individual can be in the collective effort. These functions can be selected depending on the dynamics of the problem that is being assessed. Several examples of situations and the type of production functions that capture their dynamics are presented in ([12],Ch 4),[46]. Second, the motivation function can be designed to study common effects of motivation gain or loss that shape voluntary cooperation in the community. Third, reputation and reciprocity patterns observed in the community can be included in the model [47]. The network topology can be designed to characterize the interaction patterns in the community. For example, small communities exhibit simple patterns such as centralized or centralized relations in their interactions [48], while large-scale communities can have more complex patterns [49].

## Conclusion

We characterized the dynamical process of completing a task as the result of the repeated contribution of participating individuals in a community. The relationship between the participatory action and the contribution toward the task completion is described by a production function. The process of completing the task involves only costs associated with participation. Therefore, an individual who decides to participate has to have enough motivation to engage in completing the task. The social dilemma emerges when no community member has enough motivation to assume the costs of participation. We showed that the ability of the individuals in the community to communicate can allow for the emergence of cooperation through the development of reputation and indirect reciprocity. We introduced the concept of relative reputation, defined as an individual’s reputation with respect to another individual’s point of view, leading to participation through reciprocity. Also, we propose an interaction strategy where the available participation load is locally distributed between the individuals. This strategy seeks to distribute the available task participation load so that the individuals have the same relationship between motivation and participation costs. here, individuals who are more willing to assume the participation costs are more likely to take higher loads of participation than those with lower motivation.

We used concepts of stability analysis in dynamical system theory to perform a mathematical analysis of the proposed model, and also we conducted simulations to observe the qualitative behavior of the community for different values of the parameters. We showed the conditions that guaranteed the completion of the task in the long term, and also we showed through simulations that the choice of the motivation and production functions, the topology of the interaction network, and the number of individuals, affect the dynamics of the task completion process. The results of the simulations showed that, in the chosen simulation scenario, relatively homogeneous communities that are sufficiently connected tend to have an equal distribution of the participation loads, allowing for shorter convergence times of the task for decelerating production functions, and longer convergence times for accelerating production functions. In the case of a network topology with a structure where individuals have few neighbors tended to have unequal distributions of the participation load, allowing for shorter convergence times of the task for accelerating production functions, and longer convergence times for decelerating production functions.

The model presented in this paper can be extended to study additional situations that arise in the study of the dynamics of cooperation. For example, a different definition of the motivation function can be considered. Effects such as social loafing and the Köhler effect can studied using our model. Also, situations can be explored where the members of the community have to distribute their participation level among several tasks, or where there are different topologies of the interaction network, and larger scales of the community. Concepts from role theory [50] and the theory of collective behavior [43] can be connected to the concept of production function for the analysis of collective action and additional mechanisms that solve task completion and collective goods social dilemmas.

## Supporting Information

S1 Appendix Mathematical proofs and Matlab Code. It contains the proofs of Theorems 1, 2, 3, 4, Corollary 1.1, and the Matlab Code to generate Figs 4 and 7.
(PDF)

## References

[1] Rand DG, Nowak MA. Human cooperation. Trends in Cognitive Sciences. 2013;17(8):413–425. 10.1016/j.tics.2013.06.003 23856025

[2] Perc M. Phase transitions in models of human cooperation. Physics Letters A. 2016;380(36):2803–2808. 10.1016/j.physleta.2016.06.017

[3] Kollock P. Social dilemmas: The anatomy of cooperation. Annual review of sociology. 1998; p. 183–214. 10.1146/annurev.soc.24.1.183

[4] Van Vugt M, Van Lange PA, Meertens RM. Commuting by car or public transportation? A social dilemma analysis of travel mode judgements. European Journal of Social Psychology. 1996;26(3):373–395. 10.1002/(SICI)1099-0992(199605)26:3<373::AID-EJSP760>3.0.CO;2-1

[5] Frederiks ER, Stenner K, Hobman EV. Household energy use: Applying behavioural economics to understand consumer decision-making and behaviour. Renewable and Sustainable Energy Reviews. 2015;41:1385–1394. 10.1016/j.rser.2014.09.026

[6] Rothstein B. The universal welfare state as a social dilemma. Rationality and Society. 2001;13(2):213–233. 10.1177/104346301013002004

[7] Milinski M, Sommerfeld RD, Krambeck HJ, Reed FA, Marotzke J. The collective-risk social dilemma and the prevention of simulated dangerous climate change. Proceedings of the National Academy of Sciences. 2008;105(7):2291–2294. 10.1073/pnas.0709546105 18287081 PMC2268129

[8] Jacquet J, Hagel K, Hauert C, Marotzke J, Röhl T, Milinski M. Intra-and intergenerational discounting in the climate game. Nature Climate Change. 2013;3(12):1025–1028. 10.1038/nclimate2024

[9] Kemmis D. Community and the Politics of Place. University of Oklahoma Press; 1992.

[10] Bowles S, Gintis H, et al Reciprocity, self-interest, and the welfare state. Nordic Journal of Political Economy. 2000;26(1):33–53.

[11] Olson M. The logic of collective action. Harvard University Press; 1965.

[12] Marwell G, Oliver P. The critical mass in collective action. Cambridge University Press; 1993.

[13] Perc M, Gómez-Gardeñes J, Szolnoki A, Floría LM, Moreno Y. Evolutionary dynamics of group interactions on structured populations: a review. Journal of The Royal Society Interface. 2013;10(80):1–17. 10.1098/rsif.2012.0997 23303223 PMC3565747

[14] Axelrod R, Hamilton WD. The evolution of cooperation. Science. 1981;211(4489):1390–1396. 10.1126/science.7466396 7466396

[15] Nowak MA. Five rules for the evolution of cooperation. science. 2006;314(5805):1560–1563. 10.1126/science.1133755 17158317 PMC3279745

[16] Perc M, Szolnoki A. Coevolutionary games—a mini review. BioSystems. 2010;99(2):109–125. 10.1016/j.biosystems.2009.10.003 19837129

[17] Wang Z, Wang L, Szolnoki A, Perc M. Evolutionary games on multilayer networks: a colloquium. The European Physical Journal B. 2015;88(5):1–15. 10.1140/epjb/e2015-60270-7

[18] Xia CY, Meloni S, Perc M, Moreno Y. Dynamic instability of cooperation due to diverse activity patterns in evolutionary social dilemmas. EPL (Europhysics Letters). 2015;109(5):1–6. 10.1209/0295-5075/109/58002

[19] Milinski M, Semmann D, Krambeck HJ. Reputation helps solve the tragedy of the commons. Nature. 2002;415(6870):424–426. 10.1038/415424a 11807552

[20] Chen X, Szolnoki A, Perc M. Risk-driven migration and the collective-risk social dilemma. Physical Review E. 2012;86(3):036101 10.1103/PhysRevE.86.036101 23030974

[21] Sugiarto HS, Chung NN, Lai CH, Chew LY. Socioecological regime shifts in the setting of complex social interactions. Physical Review E. 2015;91(6):062804 10.1103/PhysRevE.91.062804 26172751

[22] Tyler TR. Why people cooperate: The role of social motivations. Princeton University Press; 2010.

[23] Van Lange PA, Joireman J, Parks CD, Van Dijk E. The psychology of social dilemmas: A review. Organizational Behavior and Human Decision Processes. 2013;120(2):125–141. 10.1016/j.obhdp.2012.11.003

[24] Ostrom E. Governing the commons. Cambridge university press; 1990.

[25] Dietz T, Ostrom E, Stern PC. The struggle to govern the commons. science. 2003;302(5652):1907–1912. 10.1126/science.1091015 14671286

[26] Homan M. Promoting community change: Making it happen in the real world. Brooks/Cole; 2010.

[27] Passino KM. Humanitarian Engineering: Creating Technologies That Help People. Bede Publishing; 2015.

[28] Heckathorn DD. The dynamics and dilemmas of collective action. American Sociological Review. 1996; p. 250–277. 10.2307/2096334

[29] Cardillo A, Petri G, Nicosia V, Sinatra R, Gómez-Gardeñes J, Latora V. Evolutionary dynamics of time-resolved social interactions. Physical Review E. 2014;90(5):052825 10.1103/PhysRevE.90.052825 25493851

[30] Meng XK, Xia CY, Gao ZK, Wang L, Sun SW. Spatial prisoner’s dilemma games with increasing neighborhood size and individual diversity on two interdependent lattices. Physics Letters A. 2015;379(8):767–773. 10.1016/j.physleta.2014.12.051

[31] Finke J, Passino KM. Local agent requirements for stable emergent group distributions. IEEE Transactions on Automatic Control. 2011;56(6):1426–1431. 10.1109/TAC.2011.2112476

[32] Giraldo LF, Passino KM. Dynamic Task Performance, Cohesion, and Communications in Human Groups. IEEE Transactions on Cybernetics. To be published;. 10.1109/TCYB.2015.2470225 26340793

[33] Pavlic TP, Passino KM. Distributed and cooperative task processing: Cournot oligopolies on a graph. IEEE Transactions on Cybernetics. 2014;44(6):774–784. 10.1109/TCYB.2013.2271776 24839060

[34] Kerr NL. Motivation losses in small groups: A social dilemma analysis. Journal of Personality and Social Psychology. 1983;45(4):819–828. 10.1037/0022-3514.45.4.819

[35] Karau SJ, Williams KD. Social loafing: A meta-analytic review and theoretical integration. Journal of personality and social psychology. 1993;65(4):681–706. 10.1037/0022-3514.65.4.681

[36] Shepperd JA. Productivity loss in performance groups: A motivation analysis. Psychological bulletin. 1993;113(1):67–81. 10.1037/0033-2909.113.1.67

[37] Shepperd JA. The desire to help and behavior in social dilemmas: Exploring responses to catastrophes. Group Dynamics: Theory, Research, and Practice. 2001;5(4):304–314. 10.1037/1089-2699.5.4.304

[38] Kerr NL, Feltz DL, Irwin BC. To pay or not to pay? Do extrinsic incentives alter the Köhler group motivation gain? Group Processes & Intergroup Relations. 2012; p. 257–268.

[39] Kerr NL, Kaufman-Gilliland CM. Communication, commitment, and cooperation in social dilemma. Journal of personality and social psychology. 1994;66(3):513 10.1037/0022-3514.66.3.513

[40] Balliet D. Communication and cooperation in social dilemmas: A meta-analytic review. Journal of Conflict Resolution. 2009;.

[41] Nowak MA, Sigmund K. Evolution of indirect reciprocity. Nature. 2005;437(7063):1291–1298. 10.1038/nature04131 16251955

[42] Kerr NL, Messé LA, Seok DH, Sambolec EJ, Lount RB, Park ES. Psychological mechanisms underlying the Köhler motivation gain. Personality and Social Psychology Bulletin. 2007;33(6):828–841. 10.1177/0146167207301020 17475617

[43] Turner R, Killian L. Collective behavior. 3rd ed Prentice-Hall; 1987.

[44] Nowak M, Highfield R. SuperCooperators: Altruism, evolution, and why we need each other to succeed. Simon and Schuster; 2011.

[45] McTaggart R. Principles for participatory action research. Adult Education Quarterly. 1991;41(3):168–187. 10.1177/0001848191041003003

[46] Heckathorn DD, Rosenstein JE. Group solidarity as the product of collective action: creation of solidarity in a population of injection drug users. Advances in Group Processes. 2002;19:37–66. 10.1016/S0882-6145(02)19003-5

[47] Yoeli E, Hoffman M, Rand DG, Nowak MA. Powering up with indirect reciprocity in a large-scale field experiment. Proceedings of the National Academy of Sciences. 2013;110(Supplement 2):10424–10429. 10.1073/pnas.1301210110 23754399 PMC3690615

[48] Katz N, Lazer D, Arrow H, Contractor N. The network perspective on small groups Theories of small groups: Interdisciplinary perspectives. 2005; p. 277–312.

[49] Ravasz E, Barabási AL. Hierarchical organization in complex networks. Physical Review E. 2003;67(2):1–7. 10.1103/PhysRevE.67.026112 12636753

[50] Biddle BJ. Recent development in role theory. Annual review of sociology. 1986; p. 67–92. 10.1146/annurev.so.12.080186.000435

