# Supplementary material for: Dynamics of Cooperation in a Task Completion Social Dilemma
Source: PLoS One. 2017 Jan 26;12(1):e0170604. doi: 10.1371/journal.pone.0170604 (PMC5270338; doi:10.1371/journal.pone.0170604)
Supplement: S1 Appendix — It contains the proofs of Theorems 1, 2, 3, 4, Corollary 1.1, and the Matlab Code to generate Figs 4 and 7. (PDF) [file pone.0170604.s001.pdf]

S1 Appendix: Dynamics of Cooperation in a Task Completion Social Dilemma

Luis Felipe Giraldo and Kevin M Passino

1 Proofs Theorems and Corollary

1.1 Proof Theorem 1

This proof is based on concepts from Lyapunov stability theory in the analysis of discrete nonlinear systems [1]. We will provide sufficient conditions such that the trajectories of z converge to $\bar{z}$ in Eq (2).
First, we define the variable $\hat{z}(t) = \bar{z} - z(t)$. Eq (2) can be rewritten as

$$\hat{z}(t+1) = [1 - \phi h(p(t))]\, \hat{z}(t) \tag{S1}$$

where $0 \leq \hat{z}(0) \leq \bar{z}$. Since $\phi h(p(t)) \in [0, 1)$, we know that $0 \leq z(t) \leq \bar{z}$ for all $t \geq 0$ if $z(0) \leq \bar{z}$. From [1, Theorem 1], if there exists a strictly increasing function ψ, with $\psi(0) = 0$, such that $V(\hat{z}(t+1)) - V(\hat{z}(t)) \leq -\psi(|\hat{z}|)$ for all $t \geq 0$, with $V(\hat{z}(t)) = \frac{1}{\phi}|\hat{z}(t)|$ (Lyapunov function candidate), then the point $\hat{z} = 0$ is asymptotically stable. That is, the trajectories of $\hat{z}$ with initial conditions $0 \leq \hat{z}(0) \leq \bar{z}$ will asymptotically converge to 0. Using Eq (S1) we obtain

$$\begin{aligned} V(\hat{z}(t+1)) - V(\hat{z}(t)) &= \frac{1}{\phi}\,|1 - \phi h(p(t))|\,|\hat{z}| - \frac{1}{\phi}\,|\hat{z}| \\ &= \frac{1}{\phi}\,||1 - \phi h(p(t))| - 1|\,|\hat{z}| \end{aligned}$$

From the assumption that $h(p(t)) \in [0, 1)$ for all $t \geq 0$, we have

$$V(\hat{z}(t+1)) - V(\hat{z}(t)) = -h(p(t))\,|\hat{z}|$$

Then, if we can find such function ψ such that $-h(p(t))\,|\hat{z}| \leq \psi(|\hat{z}|)$ for all $t \geq 0$, then $\hat{z}$ will converge to 0.

1.2 Proof Corollary 1.1

Let p^* the fixed participation load taken by the individuals. Form the previous proof, we have that

$$V(\hat{z}(t+1)) - V(\hat{z}(t)) = -h(p^*)\,|\hat{z}|$$

Since $V(\hat{z}(t)) = \frac{1}{\phi}|\hat{z}(t)|$, from [2, Theorem 3], we have then that $\hat{z}$ converges exponentially to zero.

From Eq (S1), given the initial condition $\hat{z}(0)$, the trajectory of $\hat{z}(t)$ is given by

$$\hat{z}(t) = [1 - \phi h(p^*)]^t\, \hat{z}(0)$$

Substituting $\hat{z}$ by $\bar{z} - z$, we obtain Eq (5).

1.3 Proof Theorem 2

This proof is based on Lagrange multiplier theory and the Karush-Kuhn-Tucker necessary and sufficient conditions for constrained optimization problems [3, Ch 3]. We show how the assumptions on the marginal gain functions and the formulation of the problem allows us to state the theorem.

Since U is a strictly concave function, and $\sum_{i=1}^{n} p_i = P$ is in the feasible region, we know that p^* satisfies $\sum_{i=1}^{n} p_i = P$ since every increment in p will generate an increment in U. From the Karush-Kuhn-Tucker necessary conditions, there exist $\mu^*, \lambda_i^* \geq 0$, $i = 1, \ldots, n$, such that

$$-g(p_i^*) + \mu^* - \lambda_i^* = 0, \quad i = 1, \ldots, n \tag{S2}$$

where μ^* is the Lagrange multiplier associated with the constraint $\sum_{i=1}^{P} p_i \leq P$, and λ_i^* is the Lagrange multiplier associated with the constraint $-p_i \leq 0$. Since $g_i(p_i) > 0$ for $p_i \in [0, P]$, the Lagrange multipliers satisfy $\mu^* - \lambda_i^* > 0$. If $-p_i^* > 0$, that is, the constraint is inactive, then $\lambda_i^* = 0$, and $g(p_i^*) = \mu^*$. Therefore, $g(p_i^*) = g_j(p_j^*)$ will be satisfied for every $i, j = 1, \ldots, n$ for which $p_i^*, p_j^* > 0$.

From the previous result and the assumptions on g_i as a function of p_i, we have that p^* satisfies the sufficient conditions to be a strict maximum of U over the feasible region [3, Proposition 3.2.2]. Since this is a strict concave optimization problem, this maximum is unique.

1.4 Proof Theorem 3

We show that the load distribution algorithm satisfies the conditions presented in [4] under which a load is distributed across the nodes in a network so that the marginal gain of any pair of nodes, quantified in this case by $g_i(p_i(t))$, is equalized. In this proof, we refer to the marginal gain $g_i(p_i)$ as the *suitability* of node i given p_i to follow the notation in [4].

In [4, Theorem 3.4], it is shown that if some conditions are satisfied, then the rule in Eq (14) will lead to a distribution of the total available load P so that the nodes' suitability in the network is equalized. In our theorem, we have the assumptions that the network is connected, and the marginal gain is positive, and decreasing and differentiable with respect to p_i. These assumptions, along with lines 6 to 9 in the algorithm, satisfy the conditions (a), (b), (c) in Assumption 1 in [4]. Condition (d) states that the total participation load P must be greater that some bound that can be unnecessarily conservative. In our problem, since we have shown the relationship between the optimization problem in Theorem 2 and the distribution algorithm, it is enough to have a value of P so that the optimization problem has an optimal distribution whose entries are (strictly) greater than zero.

From [4, Theorem 3.4], these four conditions guarantee that the transference of load between nodes leads to a state that is invariant where the suitability between every pair of nodes associated with the community members is the same, converging at an exponential rate. If the optimization problem in Theorem 2 has a maximum with zero entries, then the algorithm will equalize the suitability of those nodes associated with the positive entries.

1.5 Proof Theorem 4

This proof is a straightforward result from Theorem 1. Since at least one individual satisfies $g_i(p_i, t) > 0$ for $p_i \in [0, P]$, the distribution algorithm will assign positive amounts of participation load based on the current marginal gains at each instant of time. Let $\eta = \min_{t \geq 0} \{h(p(t))\}$, where we know that $\eta > 0$. Then, we obtain

$$h(p(t))|\bar{z} - z(t)| \geq \eta |\bar{z} - z(t)|$$

The right-hand side of this equation is a strictly increasing function of $|\bar{z} - z(t)|$. Then, from Theorem 1, we have that z converges to $z(t)$.

2 Implementation Details

2.1 Parameters in Monte Carlo Simulations

Total participation load P	1
Completed task state $\bar{z}$	1
Scale of production function ϕ	0.25
Maximum reputation $\bar{r}$	3
Load passing rate θ	0.005
Reciprocity influence $\delta_i = \delta$	1
Costs of participation (linear function) c_i	$c_i \sim$ Uniform$[0.5, 3]$
Motivation γ_i	$\gamma_i = c_i/P$, $\gamma_j = 0$, $i \neq j$, and i are randomly selected
Reputation rate of change λ_i	$\lambda_i \sim U[0.05, 1]$
Initial reputation $r_{ij}(0)$ and task variable $z(0)$	0
Production function $h(p)$	$h(p) = \min\left\{\sum_{i=1}^{n} \frac{p_i^\alpha}{5P^\alpha}, 1\right\}$

2.2 Matlab Code: Plots in Figure 4

```
function [] = gainBalancing()

clc, close all;

%—— Parameters of the model
theta = 0.001;   % Unit load to be passed at each iteration
Tmax = 15;       % Maximum number of iterations

% Adjacency matrix of line topology
A = logical([0 1 0;
             1 0 1;
             0 1 0]);
n = size(A,1);          % Number of individuals

% Gains
b=(3/2)*[2;1.5;3]; % Benefits
c=[3;2;4];         % Costs
P=1;               % Maximum participation load


%—— Initial conditions
% Maximum value of p where g is positive
p = [P 0 0]';


%—— Main loop
mu(1) = levelOfSatisfaction(p,b,c);
g=netGain(p,b,c);
for k=1:Tmax
    p(:,k+1)=p(:,k);
    g(:,k+1)=netGain(p(:,k+1),b,c);

    %———— Assign loads to pass between individuals
    for i=1:n
        % Create set Si
        Si = zeros(1,n);
        for j=1:n
            if A(i,j)==1
                if g(j,k+1)>g(i,k+1)
                    Si(1,j) = g(j,k+1);
                end;
```

```
            end;
        end;

        if any(Si)
            mSi=max(Si);
            jm = find(Si==mSi);

            %--- Load to pass from i to jm
            L = generateLoad_i(p(i,k+1),b(i),c(i),...
                p(jm,k+1),b(jm),c(jm),theta);

            p(i,k+1) = p(i,k+1) - sum(L);
            g(i,k+1) = netGain(p(i,k+1),b(i),c(i));

            p(jm,k+1) = p(jm,k+1) + L;
            g(jm,k+1) = netGain(p(jm,k+1),b(jm),c(jm));
        end;
    end;

    mu(k+1) = levelOfSatisfaction(p(:,k+1),b,c);
end;


%% Plots
figure,
subplot(311),hold on
plot(g(1,:),'b','linewidth',2),
plot(g(2,:),'r--','linewidth',2),
plot(g(3,:),'g-.','linewidth',2),
hold off
xlabel('Time steps, $t$','Interpreter','latex','fontsize',16),
ylabel('${g}_i(t)$','interpreter','latex','fontsize',16)
axis([1 Tmax 0 5])
legend({'Individual 1','Individual 2','Individual 3'}, 'Orientation', 'horizontal')
legend boxoff
box off

subplot(312),hold on
plot(p(1,:),'b','linewidth',2),
plot(p(2,:),'r--','linewidth',2),
plot(p(3,:),'g-.','linewidth',2),
hold off
xlabel('Time steps, $t$','interpreter','latex','fontsize',16),
ylabel('$p_i(t)$','interpreter','latex','fontsize',16)
axis([1 Tmax 0 1])
legend({'Individual 1','Individual 2','Individual 3'}, 'Orientation','horizontal')
legend boxoff
box off

subplot(313),
plot(mu,'linewidth',2),
xlabel('Time steps, $t$','interpreter','latex','fontsize',16),
ylabel('$U(p_i(t))$','interpreter','latex','fontsize',16)
axis([1 Tmax 1.4 3])
box off


%---------------------------------------------------------------
% Level of satisfaction or utility of the community
function [mu] = levelOfSatisfaction(p,b,c)
mu = sum(b.*p - 0.5*c.*(p.^2));

%---------------------------------------------------------------
% Marginal gain
function [g] = netGain(p,b,c)
g = b - c.*p;
```

```
%———————————————————————————————
% Generate load to pass
function [Lij] = generateLoad_i(pi,bi,ci,pj,bj,cj,theta)
nSi = length(pj);

nL = 500;
L = pi*rand(nL,nSi);
gi = netGain(pi,bi*ones(nL,1),ci*ones(nL,1));
gim = netGain(pi-sum(L,2),bi*ones(nL,1),ci*ones(nL,1));


S = (gim >=0);
for k=1:nSi
    gj(:,k) = netGain(pj(k),bj(k)*ones(nL,1),cj(k)*ones(nL,1));
    gjp(:,k) = netGain(pj(k)+L(:,k),bj(k)*ones(nL,1),cj(k)*ones(nL,1));

    S = S & ( (gim <= gjp(:,k)) & (gjp(:,k) <= (gj(:,k) - theta*(gj(:,k)-gi)))...
             & (gjp(:,k) >= 0));
end;
Lij = L(S,:);
if isempty(Lij),
    Lij=0;
else
    %Lij = max(Lij);
    Lij = Lij(1,:)';
end;
```

2.3 Matlab Code: Simulation in Figure 6

```
function [] = taskCompletion()
clc, close all;

rng(32) % Fix seed for pseudorandom number generator


%—— Parameters of the balancing algorithm
theta = 0.005;   % Unit load to be passed at each iteration

% Adjacency matrix
A= logical([0 1 1 0 0;
            1 0 0 0 0;
            1 0 0 1 1;
            0 0 1 0 0;
            0 0 1 0 0]) ;
n = size(A,1);  % Number of agents

%—— Parameters of the model
P=1;                        % Maximum participation load
b = [3;zeros(n-1,1)];      % Motivation
c = [2;0.75;1;1.5;2.5];        % Cost per unit of participation load
zbar = 1;                  % Maximum task completion value
rbar = 3;                   % Maximum reputation
phi = 0.25;                  % Scale factor production function

% Contribution to reputation building
delta = A.*(ones(n)-diag(ones(1,n)));

% Reputation forgetting factor
lambda = 0.05*rand(n)+0.05;
lambda = A.*(lambda - diag(diag(lambda)));


%—— Initial conditions
z=0;              % Task completion
rij = zeros(n);   % Reputation
reciprocity = sum(rij.*delta,2); % Reciprocity

% Maximum value of p where g is positive
```

```
gainz = b+reciprocity;

p = [P;zeros(n-1,1)];
g=netGain(p,gainz,c,P);

mu(1) = levelOfSatisfaction(p,b,c);


%--- Main loop
Tmax = 120;  % Maximum number of iterations
for k=1:Tmax
    %------- Compute motivation + reciprocity
    reciprocity = sum(rij(:,:,k).*delta,2); % Reciprocity
    gainz = b+reciprocity;                  % Benefits

    %------- Update task completion
    z(k+1)= z(k) + phi*prodFunct(p(:,k),P)*(zbar-z(k));

    %------- Update reputation
    for i=1:n
        rij(i,:,k+1) = rij(i,:,k).*(1-lambda(i,:))...
            + lambda(i,:).*p(:,k)'*rbar/P;
    end;


    %------- Assign loads to pass between individuals
    p(:,k+1)=p(:,k);
    g(:,k+1)=netGain(p(:,k+1),gainz,c,P);
    for i=1:n
        % Create set Si
        Si = zeros(1,n);
        for j=1:n
            if A(i,j)==1
                if (g(j,k+1)>g(i,k+1))
                    Si(1,j) = g(j,k+1);
                end;
            end;
        end;

        if any(Si)
            mSi=max(Si);
            jm = find(Si==mSi);

            %--- Load to pass from i to jm
            L = generateLoad_i(p(i,k+1),gainz(i),c(i),...
                p(jm,k+1),gainz(jm),c(jm),theta,P);

            p(i,k+1) = p(i,k+1) - sum(L);
            g(i,k+1) = netGain(p(i,k+1),gainz(i),c(i),P);

            p(jm,k+1) = p(jm,k+1) + L;
            g(jm,k+1) = netGain(p(jm,k+1),gainz(jm),c(jm),P);
        end;
    end;

    mu(k+1) = levelOfSatisfaction(p(:,k+1),gainz,c);
end;

%% Plots
%---- Plot marginal gain functions
subplot(311),
plotinfo=plot(0:Tmax-1,g(:,1:Tmax)','LineWidth',2);
ylabel('${g}_i(t)$', 'interpreter','latex','fontsize',16)
box off
hold on,
markers = '*ov+s';
for i=1:n
  plot((1:7:Tmax)-1,g(i,1:7:Tmax),markers(i),'Color',plotinfo(i).Color)
```

```
end;
for i=1:n
  phandle(i) = plot(0,0,[markers(i) '-'],'Color',plotinfo(i).Color);
end;
ax = gca;
ax.FontSize=14;
hold off

legend(phandle,{...
    '${g}_1$','${g}_2$','${ g}_3$','${ g}_4$','${ g}_5$'},...
    'interpreter','latex','fontsize',14,'orientation','horizontal')
legend boxoff


% Plot weights
subplot(312),
plotinfo=plot(0:Tmax-1,p(1:n,1:Tmax)','LineWidth',2);
hold on,
markers = '*ov+s';
for i=1:n
  plot(0:7:Tmax-1,p(i,1:7:Tmax)',markers(i),'Color',plotinfo(i).Color)
end;
for i=1:n
  phandle(i) = plot(0,0,[markers(i) '-'],'Color',plotinfo(i).Color);
end;

ax = gca;
ax.FontSize=14;
box off;
ylabel('$p_i(t)$', 'interpreter','latex','fontsize',16)
hold off

legend(phandle,{'$p_1$','$p_2$','$p_3$','$p_4$','$p_5$'},...
    'interpreter','latex','fontsize',14,'orientation','horizontal')
legend boxoff


% Plot task completion variable
subplot(313),
plot(0:Tmax-1,z(1:Tmax),'linewidth',2)
ylabel('$z(t)$', 'interpreter','latex','fontsize',16)
ax = gca;
ax.FontSize=14;
ax.YTick = [0 zbar];
ax.YTickLabel = {'0','$\bar z$'};
ax.TickLabelInterpreter= 'latex';
xlabel('Time steps, $t$', 'interpreter','latex','fontsize',16)
box off


%-----------------------------------------------------------
function [h] = prodFunct(p,P)

% Production function
n=length(p);

w=[1 2 2 1 1]';
alpha = [0.2 0.2 0.2 0.2 0.2];
w=P*w./sum(w);
h=0;
for i=1:n
    h = h + w(i)*(p(i)^alpha(i));
end;


%-----------------------------------------------------------
% Level of satisfaction or utility of the community
function [mu] = levelOfSatisfaction(p,b,c)
```

```
mu = sum(b.*p - 0.5*c.*(p.^2));

%----------------------------------------------------------------
% Marginal gain
function [g] = netGain(p,b,c,P)
g = b - c.*p;
g((b./c)<P)=0;

%----------------------------------------------------------------
% Generate load to pass
function [Lij] = generateLoad_i(pi,bi,ci,pj,bj,cj,theta,P)
nSi = length(pj);

nL = 750;
L = pi*rand(nL,nSi);
gi = netGain(pi,bi*ones(nL,1),ci*ones(nL,1),P);
gim = netGain(pi-sum(L,2),bi*ones(nL,1),ci*ones(nL,1),P);


S = (gim >=0);
for k=1:nSi
    gj(:,k) = netGain(pj(k),bj(k)*ones(nL,1),cj(k)*ones(nL,1),P);
    gjp(:,k) = netGain(pj(k)+L(:,k),bj(k)*ones(nL,1),cj(k)*ones(nL,1),P);

    S = S & ( (gim <= gjp(:,k)) & (gjp(:,k) <= (gj(:,k) - theta*(gj(:,k)-gi)))...
              & (gjp(:,k) >= 0));
end;
Lij = L(S,:);
if isempty(Lij),
    Lij=0;
else
    %Lij = max(Lij);
    Lij = Lij(1,:)';
end;
```

References

[1] Kalman R, Bertram J. Control System Analysis and Design Via the Second Method of Lyapunov: II-Discrete-Time Systems. Journal of Basic Engineering. 1960;82(2):394–400.

[2] Burgess KL, Passino KM. Stability analysis of load balancing systems. International Journal of Control. 1995;61(2):357–393.

[3] Bertsekas DP. Nonlinear programming. 1999;.

[4] Finke J, Passino KM. Local agent requirements for stable emergent group distributions. IEEE Transactions on Automatic Control. 2011;56(6):1426–1431.
